# GaN Haeckelite Single-Layered Nanostructures: Monolayer and Nanotubes

**DOI:** 10.1038/srep17902

**Published:** 2015-12-10

**Authors:** Dulce C. Camacho-Mojica, Florentino López-Urías

**Affiliations:** 1 Advanced Materials Department, IPICYT, Camino a la Presa San José 2055, Col. Lomas 4a Sección, 78216, San Luis Potosí, México

## Abstract

Nowadays, III-V semiconductors are interesting candidate materials for the tailoring of two dimensional (2D) graphene-like structures. These new 2D materials have attracted profound interest opening the possibility to find semiconductor materials with unexplored properties. First-principles density functional theory calculations are performed in order to investigate the electronic properties of GaN planar and nanotube morphologies based on Haeckelite structures (containing octagonal and square membered rings). Optimized geometries, band-structures, phonon dispersion, binding energies, transmission electron microscopy images simulations, x-ray diffraction patterns, charge densities, and electronic band gaps are calculated. We demonstrated that GaN Haeckelite structures are stable exhibiting a semiconducting behavior with an indirect band gap. Furthermore, it was found that GaN Haeckelite nanotubes are semiconductor with a band gap nature (direct or indirect) that depends of the nanotube´s chirality and diameter. In addition, it was demonstrated that surface passivation and the interaction with hydrazine, water, ammonia, and carbon monoxide molecules can change the band-gap nature. Our results are compared with the corresponding GaN hexagonal honeycomb structures.

One of the most famous prototypes of layered materials is graphene, which is a perfect two dimensional system with a single atom thickness^1^,^2^. Its structure consists of a hexagonal honeycomb structure with carbon atoms joined to three carbon nearest-neighbor with sp^2^ bonding. Similarly to graphene, new layered materials such as metal chalcogenides^3^,^4^, metal oxides^5^,^6^, and lately III-V semiconducting compound^7^,^8^,^9^,^10^,^11^,^12^,^13^,^14^,^15^,^16^,^17^,^18^,^19^,^20^,^21^,^22^,^23^,^24^,^25^,^26^,^27^,^28^,^29^,^30^,^31^ have debuted, offering extraordinary properties on a rich diversity of morphologies. Regarding III-V nitride semiconductors, it is appreciable that they are materials of great interest due to their multiple applications in electronic devices^7^,^8^ such as photodetectors^9^,^10^, light-emitting diodes^11^ lasers^12^ and solar cells^13^. GaN, AlN and InN among others crystallize in a Wurtzite structure exhibiting a direct band gap in Γ point^14^,^15^. For instance, GaN with a band gap of 3.2 eV presents UV light-emitting. While the bulk system has been widely studied, few studies had been performed in ultrathin films of few nanometers in thickness.

Freeman *et al.*^16^ investigated using first-principles calculation ultrathin films of AlN, BeO, GaN, SiC, ZnO, and ZnS. They demonstrated that ultrathin films in [0001] are non-longer polar surfaces, adopting a graphitic-like structure. More recently theoretical study reported by Wu *et al.*^17^ demonstrated that the thickness range of stable graphitic films depends sensitively on strain. Tusche *et al.*^18^ reported the growth of two-layer of ZnO (0001) on Ag(111). The authors determined by scanning tunneling microscopy characterization an atomically flat ZnO double-layer and third layer triangular clusters. Experimental evidences on AlN nanosheets were reported by Tsipas *et al.*^19^. They grew ultrathin (sub-monolayer to 12 monolayers) AlN nanosheets epitaxially by plasma assisted molecular beam epitaxy on Ag(111) single crystals. They provide evidence using electron diffraction and scanning tunneling microscopy of AlN on Ag, thus demonstrating that AlN adopts a graphite-like hexagonal structure with a larger lattice constant compared to the bulk-like Wurtzite AlN. However, monolayer and few layer materials made of GaN and other III-V semiconductors are now a challenge for the experimentalist.

More efforts concerning theoretical investigations of two dimensional systems based on III-V semiconductors have been performed hitherto. An exhaustive study on two-dimensional honeycomb structures of compounds of group III-V elements was performed by Sahin *et al.*^20^. By using density functional theory, they found that BN, AlN, GaN, InN, BP, BAs in a honeycomb structure are energetically stable. Recently, Fang-Ling *et al.*^21^ investigated the electronic and magnetic properties of C-doped GaN nanoribbons with zigzag and armchair edges. Thermomechanical properties of GaN monolayer and nanotubes were studied by Sarma *et al.*^22^. The electronic properties of semifluorinated and semihydrogenated III-V sheets from first-principles were investigated^23^,^24^,^25^,^26^. Wang *et al.*^23^ investigated structural and electronic properties of different III-V hexagonal hydrogenated monolayers. They show that the bond lengths and the cohesive energy of the hydrogenated layers are dependent of the radii of the elements. They found that all the hydrogenated III-V monolayers are wide-gap semiconductors, furthermore they demonstrated that the band gap (E_g_) decreases as the V element is heavier .i.e., E_g_(N) > E_g_(P) > E_g_(As). Ma *et al.*^24^ demonstrated that SiC, GeC, SnC, BN, AlN, and GaN honeycomb monolayers can present magnetic properties when they are semidecorated with H or F.

Yang *et al.*^27^ performed first-principles calculations of the electronic structures and investigated the optical properties of single-walled zigzag GaN NTs with Mg_Ga_-O_N_ co-doping. Their calculations showed that the Mg_Ga_-O_N_ co-doping can exist stably in GaN NTs. Zheng *et al.*^28^ investigated the electronic and magnetic properties of the perfect and vacancy defect AlN nanoribbon with both zigzag edge and armchair edge using first-principles calculations. The interaction of NH_3_ with aluminum nitride nanotubes (AlNNTs) was investigated on the basis of density functional theory calculations. Unlike the case of carbon nanotubes, it was found that the NH_3_ can be chemically adsorbed on the top of the aluminum atom of AlNNTs. The NH_3_ adsorption energy of AlNNTs is typically more than that of BNNTs^29^,^30^. Wang *et al.*^31^ showed that AlN nanostructures, such as nanocages, nanocones, nanotubes, and nanowires, can bind hydrogen in quasi-molecular form with binding energies of about 0.2 eV/H_2_. They claim that the advantage of using AlN nanostructures is that no additional metal doping is necessary and hence any difficulty with the clustering of deposited metal atoms is avoided. Due to difference in electronegativities between Al and N, Al sites remain positively charged and bind hydrogen primarily through a charge polarization mechanism. Jian Min *et al.*^32^ investigated the role of H-passivation of GaN nanoribbon edges in the magnetic properties. First-principles calculations of pristine and defected GaN nanotubes with honeycomb structure were investigated by Moradian *et al.*^33^, they found that nitrogen vacancy induces a spin-polarized ground-state. Most of the studies on III-V semiconductors layer materials are mainly focused on the graphene-like structures. Other two dimensional atomic arrangements could be stable or metastable such as the Haeckelite structure formed by combining square, pentagonal, hexagonal, or octagonal rings. These types of atomic arrangements have been widely studied in carbon materials, showing different electronic properties such as that of graphene^34^,^35^. The carbon 2D Haeckelite structures exhibit a metallic behavior. The generation of different membered rings in graphene monolayer has already been obtained via irradiation, thus artificial extended line defects and grain boundary in two dimensional materials could be created^36^,^37^,^38^. In binary systems such as BN, AlN, GaN, among others, square and octagonal rings could be combined without breaking stoichiometry (see Fig. 1). Recently, Li *et al.*^39^ demonstrated the existence of square-octagonal pairs in BN monolayers. They found grain boundaries formed by square-octagonal pairs in BN monolayer grown on Cu(111). Motivated by the experimental developments on the synthesis and characterization and theoretical studies of new 2D materials, in the present work, we propose a planar structure containing square and octagonal rings which is called Haeckelites 8–4.

In this paper, the electronic properties of GaN layers and nanotubes with Haeckelite and honeycomb structures are investigated by means of first principles density functional theory (DFT) calculations. To the best of our knowledge, investigations of GaN Haeckelite structures have not been reported hitherto. In the following, the method of calculations and results are shown.

## Computational details

Electronic calculations were performed using Density Functional Theory^40^,^41^. The generalized gradient approximation (GGA) with the PBE parametrization was chosen for the exchange-correlation functional^42^ as implemented in the SIESTA code^43^. The wave functions for the valence electrons were represented by a linear combination of pseudo-atomic numerical orbitals using a double-ζ polarized basis (DZP)^44^, while core electrons were represented by norm-conserving Troullier-Martins pseudopotentials in the Kleynman-Bylander non-local form^45^,^46^. The real-space grid used for charge and potential integration is equivalent to a planewave cut-off energy of 150 Ry. The pseudo-potentials (pp’s) were constructed from 3 and 5 valence electrons for the Ga and N ions respectively (Ga:3s^2^3p^1^, N:2s^2^2p^3^). The systems were simulated with 40 × 40 × 1 k-points and the inter-structures distance was kept to a minimum of 40 Å to avoid layers interactions. Density matrix and density matrix energy tolerances were both taken as 10^−4^ eV. In the calculations, all atoms contained in the systems are relaxed using conjugated gradient method and the total energy was calculated when the forces were converged to less than 0.04 eV/Å. The binding energy is determined by means of *E*_bind_ = *E*(system) – n*E*(Ga) – n*E*(N),where *E*(system) is the total energy of GaN nanotubes or monolayers. *E*(Ga) and *E*(N) are the total energies of isolated atoms, n represents the number of Ga or N atoms in the system. E_bind_ refers to the necessary energy to split into individual atoms from the nanotube or monolayer systems. Transmission electron microscopy images are simulated by using the SimulaTEM code^47^. The cohesion energy was calculated as follows: E_*a*_ = E_T_ – E_G_ – E_M_, where E_T_ corresponds to the energy of GaN monolayer with a gas molecule adsorbed. E_G_ and E_M_ correspond to energies of isolated GaN monolayer and gas molecule respectively. The counterpoise correction for the basis set superposition error was applied for all the systems and considered in the energy calculations^48^. Negative values of E_a_ mean that the molecule is absorbed by GaN monolayer.

## Results and Discussion

**Figure 2:**
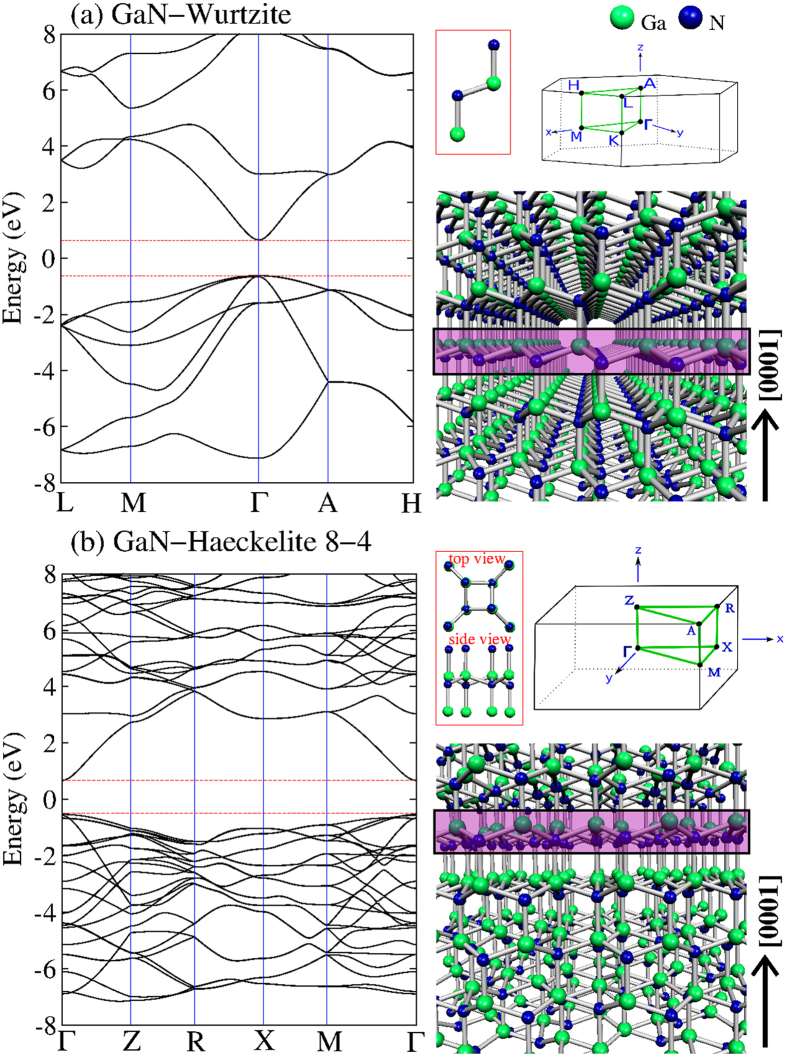
Band-structure calculations and the corresponding relaxed structures of bulk phase of GaN. The most symmetry points of the first Brillouin zone are shown. **(a)** GaN in Wurtzite structure and **(b)** Haeckelite structure with 8–4 rings. Notice that both systems exhibit a direct band-gap at Γ-point. When the isolated single or few layers are relaxed (see the single layer enclosed area in the structure models), the systems become planar.

Results on band-structure and the optimized structures of bulk for the Wurtzite and Haeckelite crystals are depicted in Fig. 2a,b respectively. For the Wurtzite structure, it was found an interatomic distance d_GaN_ of 2.02 Å with a binding energy of −4.037 eV, showing a direct gap at Γ-point of 1.37 eV. The structure in Fig. 2b was obtained by relaxing the Haeckelite structure which consists of stacking layers in the [0001] crystallographic direction, such that Ga atoms site on nitrogen atoms and vice versa. After geometry relaxation the structure is no longer a planar structure, and extra bonds along the [0001] are favored with d_GaN_ = 2.02 Å, the binding energy yields −3.892 eV which is close to that obtained from the Wurtzite structure. It was also found that the Haeckelite-like structure exhibit a direct band gap at Γ-point of 1.2 eV. From these structures (Wurtzite and Haeckelite-like) were built the 2D systems, thus the monolayers were obtained by cutting the crystal Wurtzite-type or Haeckelite structure perpendicular to the c-axis, [0001] direction (see the enclosed layers in Fig. 2). After geometry optimization on the single layer systems, the monolayers (Haeckelite and honeycomb) become flat as was previously reported for GaN and others III-V compounds with a honeycomb structure^20^. It was found that the binding energy yields −3.578 and −3.376 eV per atom for the hexagonal and Haeckelite monolayer respectively. Beyond three layers, it was found that after structure relaxation, the Haeckelite structure is no longer a planar system. Previous first-principles calculations demonstrated that up to 10 layers of GaN exhibit a honeycomb structure^17^.

**Figure 3:**
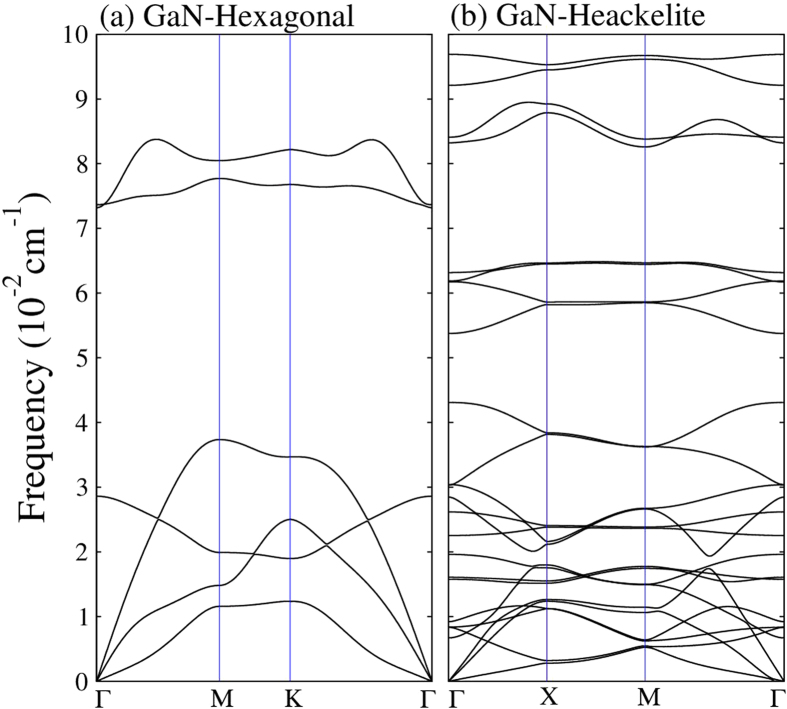
Vibration frequencies of phonon modes of GaN monolayers (a) Hexagonal and (b) Haeckelite. For both monolayers when K goes to zero, the acoustic modes LA and TA are linear.

**Figure 4:**
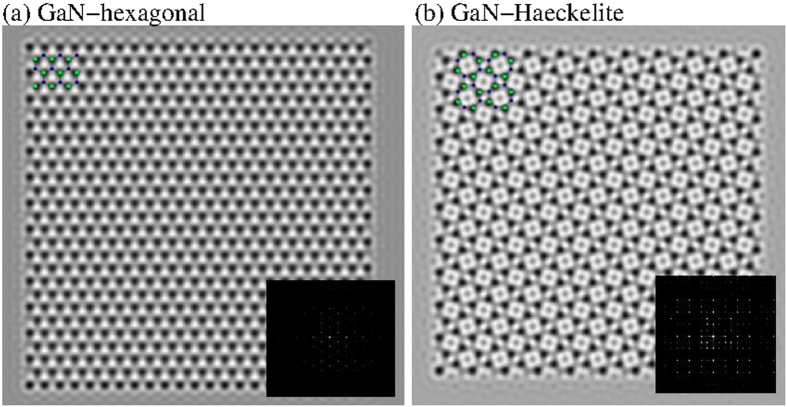
Simulated high resolution micrographs of GaN monolayers (a) hexagonal and (b) Haeckelite. The insets show the corresponding diffraction patterns. The Ga atoms are located at the dark spots.

We have also calculated the phonon modes for the Haeckelite monolayer and honeycomb structures (see Fig. 3). Our results for the phonon dispersion curves for the honeycomb monolayer are in agreement with previous calculations^20^. If there are p atoms in the unit cell, there are 3p branches to the dispersion relation: 3 acoustical modes and 3p–3 optical modes^49^. Thus the hexagonal monolayer with two atoms in the unit cell contains six modes: one longitudinal acoustic (LA) one transversal acoustic (TA) and one out-of-plane acoustic (ZA) and 3 optical modes. For the Haeckelite monolayer with 8 atoms in the primitive cell we found 24 branches one LA one TA one ZA and 21 optical modes, furthermore when k→0 the behavior of LA and TA is linear while ZA is quadratic. For both monolayers, it was found that all calculated frequencies in the Brillouin zone are positive, thus confirming the stability of the Haeckelite monolayer. Simulation of high resolution micrographs and the corresponding diffraction patterns for both monolayers are shown in Fig. 4; the dark spots in the images correspond to Ga atoms location.

**Figure 5:**
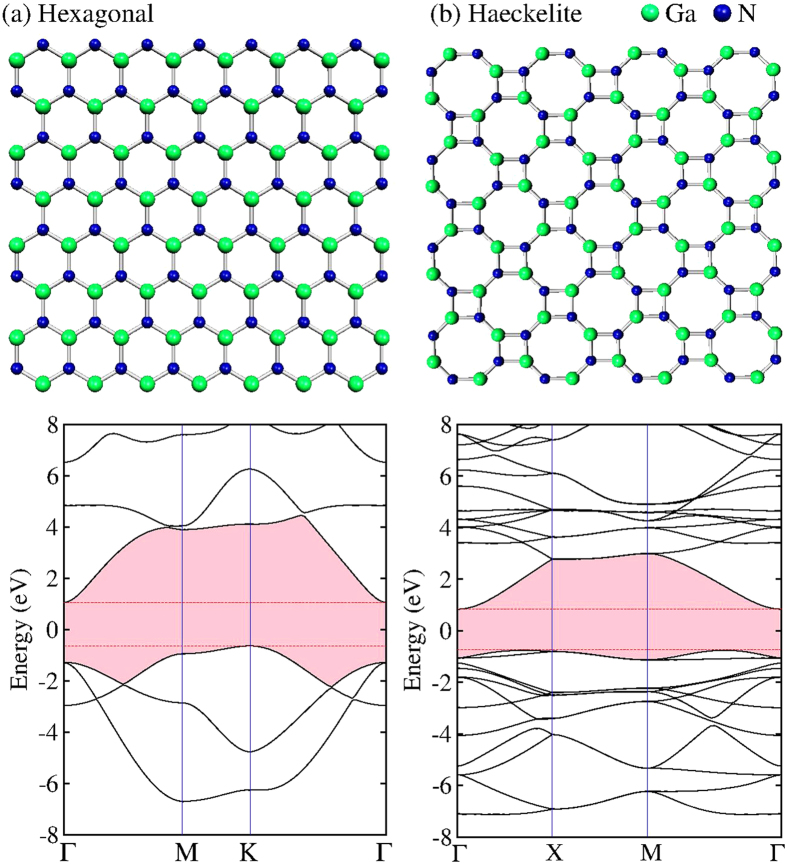
Optimized structures and band-structure of two dimensional structures of GaN. **(a)** Hexagonal honeycomb structure with only hexagonal rings, **(b)** Haeckelite structure with octagonal and square rings.

**Figure 6:**
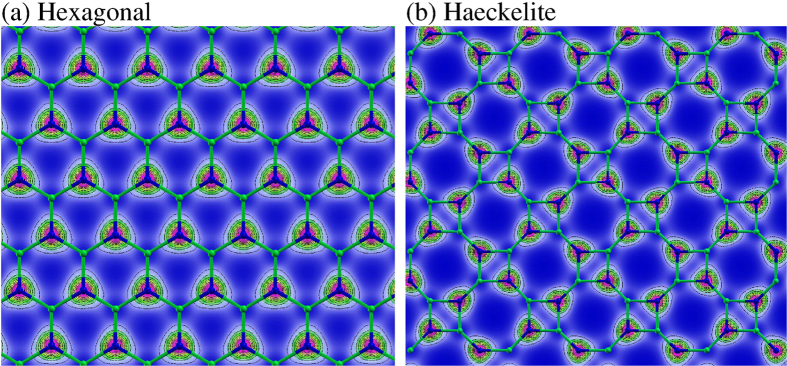
Charge density slice and contours plot for two dimensional relaxed GaN structures. **(a)** Hexagonal graphene-like structure, **(b)** Haeckelite structure. Blue regions indicate less charge.

In Fig. 5, the geometry and the band structure of the single-layer systems are depicted. The hexagonal or honeycomb structures are shown in Fig. 5a; notice that only hexagonal rings with alternated Ga and N atoms are present. The interatomic distance between Ga and N is of d_Ga-N_ = 1.92 Å. Our results are in agreement with GaN honeycomb monolayer calculations previously performed by Sahin *et al.*^20^. Figure 5b displays the Haeckelite structure with octagonal and square rings, here we found two different interatomic distances, d_Ga-N_ = 1.85 Å (bond belonging to octagons) and d_Ga-N_ = 1.95 Å (bond belonging to squares). Band-structure calculations results for the honeycomb GaN monolayer are shown in Fig. 5a. An indirect band gap of 1.68 eV was observed. The valence band maximum (VBM) is at K-point, whereas the conduction band maximum (CBM) is at Γ-point. The direct band-gap observed at Γ point is about 2.33 eV. The band-structure plot for the Haeckelite structure exhibits an indirect band gap of 1.6 eV, here the CBM is located at gamma point whereas the VBM is set in two different points (see Fig. 5b). Figure 6 displays the charge density plot of the two dimensional GaN structures. The charge distribution indicates that an electron excess is located at nitrogen atoms, whereas charge deficiency is observed in Ga atoms. Notice that Ga and N isolated atoms exhibit 3 and 5 electrons respectively. Mulliken analysis of the electronic charge (Q) indicates that in hexagonal structure the Q(N) = 5.525 and Q(Ga) = 2.475 with a notable charge transfer from Ga to N atoms. In Haeckelite structure Q(N) = 5.5 and Q(Ga) = 2.5.

**Figure 7:**
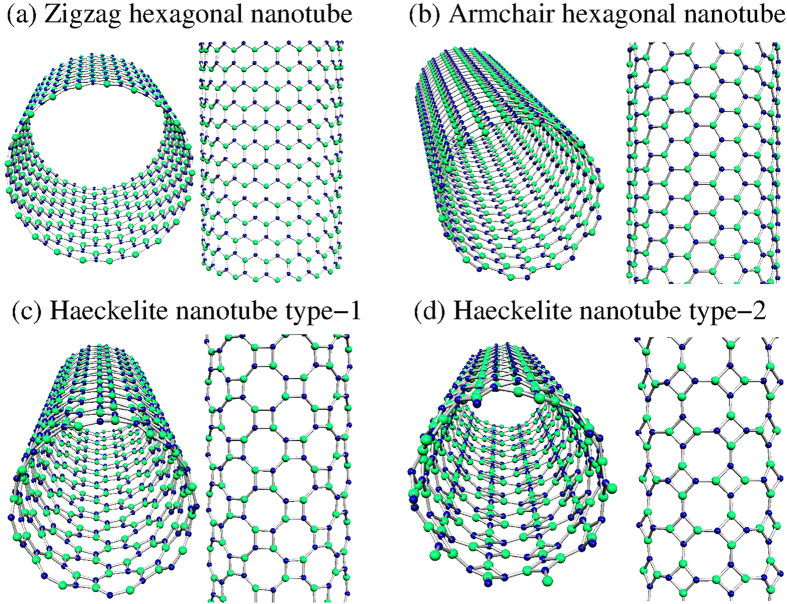
Single walled GaN nanotubes. Hexagonal honeycomb nanotubes with **(a)** zigzag and **(b)** armchair chiralities. Haeckelite nanotubes are depicted in **(c,d)** built in two different crystallographic directions labeled by type-1 (the layer is rolled up along the x-axis) and type-2 (the layer is rolled up along the y-axis). All these structure were relaxed demonstrating to be stable.

**Figure 8:**
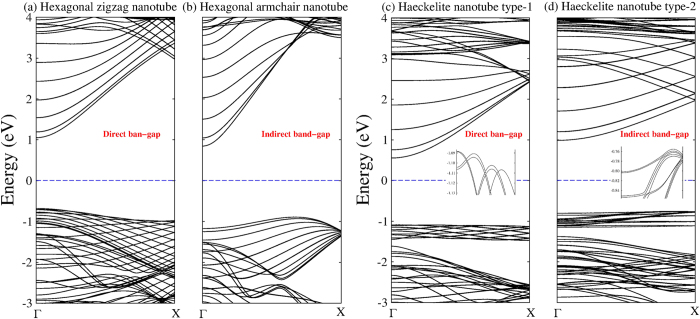
Band-structure calculations of GaN nanotubes. Hexagonal honeycomb nanotubes with **(a)** zigzag and **(b)** armchair chiralities. Haeckelite nanotubes are depicted in **(c,d)** which are built in two different crystallographic directions labeled by type-1 and type-2. Notice that the band-gap nature depends strongly of the nanotube´s chirality. Hexagonal zigzag and Haeckelite type-1 nanotubes exhibit a direct band gap at gamma point. Insets in **(c,d)** show a close up around the VBM from Γ to X. The diameters of GaN nanotubes are 22.10, 18.18, 16.57, and 14.25 Å in **(a)**, **(b)**, **(c,d)** respectively.

**Figure 9:**
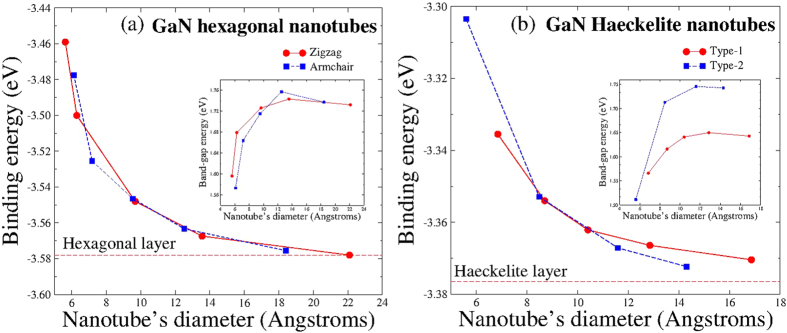
Binding energy (eV) of GaN nanotubes as (a) function of the diameter. **(b)** Hexagonal honeycomb nanotubes (zigzag and armchair) and **(b)** Haeckelite nanotubes (type-1 and type-2). The horizontal dashed lines correspond to the energy of the monolayers. The insets show the electronic band gap. Notice that all structures present a semiconducting behavior.

Regarding GaN nanotubes, they were built using the honeycomb and Haeckelite monolayers. The structure of zigzag and armchair nanotubes built from the honeycomb monolayer are shown in Fig. 7a,b respectively. The interatomic distance between Ga and N first nearest neighbors is about 1.914 Å. Figure 7c,d depicts the structure of Haeckelite nanotubes labeled by type-1 and type-2. In type-1 the squares are oriented along the nanotube axis, whereas in type-2 the squares are rotated by 45^o^ respect to the nanotube axis. In both types of Haeckelite nanotubes, two different interatomic distances are found (1.956 Å for bonds in square rings and 1.862 Å for bond in octagonal rings). Notice that in both structures (hexagonal and Haeckelite nanotubes), the interatomic distance changes are almost negligible when compared with the corresponding planar structure. All considered GaN nanotubes display a semiconducting behavior. Interestingly, band-structure calculations revealed that zigzag nanotubes exhibit a direct band-gap and armchair nanotubes present an indirect band-gap (see Fig. 8a,b) in accord with previous calculations^50^,^51^,^52^,^53^. Results for Haeckelite nanotubes are depicted in Fig. 8c,d. Notice that also Haeckelite nanotubes exhibit a chirality dependence of the band gap; nanotubes type-1 exhibit a direct band-gap whereas nanotubes type-2 exhibit an indirect band gap. Figure 9 displays the binding energy as a function of the nanotube diameter. It was found that the energetic stability of GaN nanotubes depends on the nanotube diameter. In general, narrow nanotubes are less stable and the armchair nanotubes (honeycomb and Haeckelite) are more stable than the zigzag nanotubes. The electronic band gap as a function of the diameter can be seen in the insets of Fig. 9. Notice that narrow nanotubes (diameter <8 Å) exhibit smaller band gap energy than the corresponding monolayer band gaps. For larger diameters (at least the considered in our calculations) the band gap becomes greater than the corresponding monolayer band gaps. In the limit of infinite nanotube diameter, it is expected to obtain a band gap close of that observed for the monolayer.

In order to test the thermal stability of GaN layer materials, molecular dynamics (MD) calculations were carried out on single layer based on hexagonal and Haeckelite structures. We use a time step of 1 fs for the integration of the equations of motion, for a minimum of 1000 steps. The temperature of 300 K is controlled with the NoseParrinelloRahman thermostat, using a Nosé mass of 10.0 Ry·fs^2^. We found that at 300 K, both systems (hexagonal and Haeckelite) remains as planar structures. The interatomic distances have been slightly increased when compared with calculation at 0 K. However, the systems keep the indirect band-gap semiconductor behavior. We have also performed additional calculations incorporating the spin orbit coupling within the GGA approximation and fully-relativistic approach using a plane wave basis as implemented in the Quantum-ESPRESSO package^54^. Our results for the GaN honeycomb monolayer indicate an indirect band gap of 2.15 eV, furthermore a splitting energy in the VBM at Γ point is observed (see figure S1 in supplementary information). Regarding the GaN Haeckelite 8–4 structure, it was found that the spin-orbit coupling effect does not play an important role.

The Haeckelite structure could emerge as an extended line defect formed by 8–4 rings connecting two shifted honeycomb layer, also extended line defect could join two different layered materials, for instance GaN-AlN, thus producing a natural grain boundary. The possibility to turn the band-gap from indirect to direct and manipulate the magnitude via chemical doping and defects make these materials interesting to photoluminescence devices. Several theoretical reports have modified the band gap from indirect to direct by passivating with hydrogen or fluorine^23^,^24^,^25^. In this context, we have performed calculations of GaN Haeckelite structure passivating the nitrogen atoms with hydrogen, and gallium with fluorine. We have modified the GaN Haeckelite monolayer with H and F atoms, four possible atomic arrangements were considered, (1) Both atoms (Ga and N) were passivated with H (HGaNH), (2) Both atoms (Ga and N) were passivated with F (FGaNF), (3) Ga was passivated with H and N with F (HGaNF), and (4) Ga passivated with F and N with H (FGaNH). We have also performed calculations by considering only the honeycomb structure. Our results revealed that both monolayers (Haeckelite and hexagonal) are no longer a planar system exhibiting in some cases a direct band-gap (see figure S2 and figure S3 supplementary information). Band-structures plots for passivated hexagonal GaN monolayer can be seen in figure S4 supplementary information. Table 1 summarizes the electronic properties of the passivated monolayers. Almost all passivated monolayers yielded a direct band gap. In general, the gap increased with the passivation and charge transfer from GaN monolayer to H or F atoms was observed. A detailed analysis of the orbital occupation provided information on the bonding nature, it was found that non passivated monolayers exhibit occupied out of plane p_z_ orbitals in N atoms whereas passivated monolayers present occupied in plane p_x_ and p_y_ orbitals in N atoms.

We have also performed calculations on the adsorption energy of different molecules ((Hydrazine (N_2_H_4_), water (H_2_O), carbon monoxide (CO), and ammonia (NH_3_)) on the hexagonal and Haeckelite monolayers. Table 2 summarizes the adsorption energy, the charge transfer from the molecule to GaN monolayer or vice versa, the minimal distance between the monolayer and the molecule, and the band gap (direct or indirect is also indicated). It was observed that hydrazine and ammonia molecules exhibited the lowest adsorption energies indicating a better stability of these molecules on the monolayers. For the honeycomb monolayers, the electronic band-gaps become direct whereas the Haeckelite structure always exhibited an indirect band gap. In general, the molecules remain stable on the GaN monolayers which are attached via electrostatic interactions. The electronic band gap modification (from indirect to direct) by attaching different molecular species to the GaN monolayers could be important for photoluminescence devices. Furthermore, the values of the adsorption energy suggest that our GaN monolayers could serve as chemical sensors.

In summary, GaN monolayer and nanotubes demonstrated to be stable in Haeckelite structures and energetically competitive with the corresponding honeycomb structures. We have preliminary results (not shown here) on other materials such as AlN, BP, GeC, SiC, BeO, C, InN, SnC, BN, Si, ZnO among others which demonstrated to be also stable in a Haeckelite structure. It is important to mention that BP, GeC, and SiC exhibit a direct band gap in a Haeckelite structure. Contrary, AlAs, BAs, Be, BeC, GaAs, GaP, ZnS materials among others are not stable in a Haeckelite arrangement.

## Conclusions

We have investigated the electronic properties of GaN planar and nanotube structures with honeycomb (only hexagonal rings) and Haeckelite structure (square and octagonal rings). All calculations were performed in the framework first principles density functional theory. The single layer GaN Haeckelite structure results revealed to be stable exhibiting an indirect band-gap. Regarding the GaN nanotube Haeckelite structures, results revealed that these are stable with an electronic band-gap nature (direct or indirect) dependent of the nanotube chirality. More studies regarding defects (vacancies, grain boundaries), chemical doping, and optical properties deserve to be addressed. Furthermore, band structure and electronic band gap calculations beyond the density functional theory calculations are needed in order to take into account the electron-hole interaction through GW corrections. We hope that our results will motivate more experimental and theoretical studies on new layered III-V semiconducting materials.

## Additional Information

**How to cite this article**: Camacho-Mojica, D. C. and López-Urías, F. GaN Haeckelite Single-Layered Nanostructures: Monolayer and Nanotubes. *Sci. Rep.* **5**, 17902; doi: 10.1038/srep17902 (2015).

## Supplementary Material

Supplementary Information

## Figures and Tables

**Figure 1:**
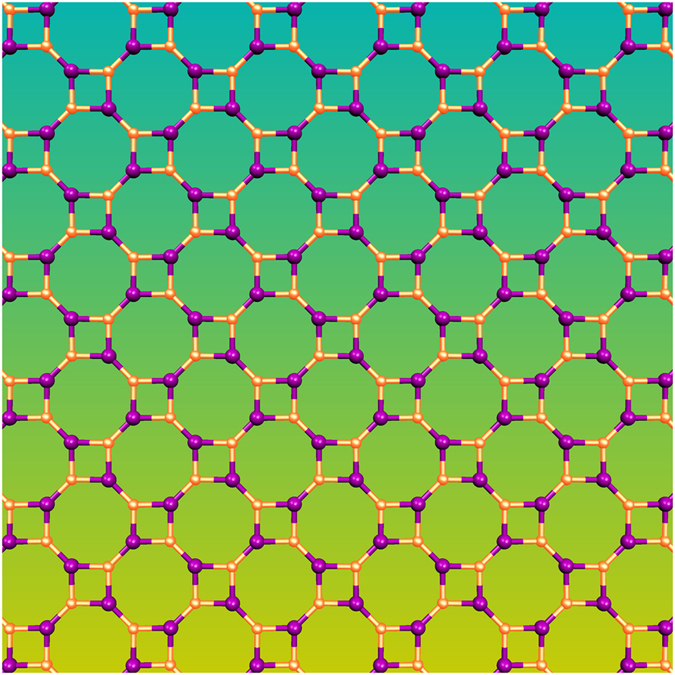
Two-dimensional Haeckelite 8–4 structure containing square and octagonal rings. In this case, the structure is decorated with two distinct types of atoms.

**Table 1:** Dulce C. Camacho-Mojica *et al.*

Structure	Band-Gap (eV)	Q(Ga)	Q(N)	Q(F)	Q(H)
Haeck-GaN	1.60 (I)	2.501	5.499	—	—
Haeck-HGaNH	3.45 (D)	2.426	5.275	—	1.164, 1.135
Haeck-FGaNF	2.23 (D)	2.310	5.280	7.320, 7.092	—
Haeck-FGaNH	2.45 (I)	2.235	5.265	7.335	1.164
Haeck-HGaNF	2.83 (D)	2.480	5.295	7.099	1.125
Hex-GaN	1.68 (I)	2.473	5.527	—	—
Hex-HGaNH	3.35 (D)	2.404	5.280	—	1.181, 1.135
Hex-FGaNF	1.54 (D)	2.281	5.307	7.316, 7.09	—
Hex-FGaNH	2.93 (D)	2.214	5.221	7.394	1.171
Hex-HGaNF	2.86 (D)	2.446	5.321	7.097	1.136

Electronic band gaps (direct (D) and indirect (I)) and the electronic charge in hexagonal GaN monolayers (Hex-GaN) and Haeckelite GaN monolayers (Haeck-GaN). Results are showed for pristine and passivated with H and F monolayers. In the last columns (Column 5 and 6), two values may appear for specific cases (when N and Ga are passivated with the same type of atom), the first value refers to the atom (H or F) -passivating Ga atoms whereas the second value refers to the atom (H or F) passivating N atoms.

**Table 2:** Dulce C. Camacho-Mojica *et al.*

System	E_a_ (eV)	*d*(Å)	Q(e)	Band-Gap (eV)
Haeck-N_2_H_4_	−0.8647	2.230	0.249	1.55 (I)
Haeck-H_2_O	−0.2905	2.008	0.025	1.58 (I)
Haeck-CO	−0.2006	3.166	0.062	1.64 (I)
Haeck-NH_3_	−0.7894	2.316	−0.006	1.66 (I)
Hex-N_2_H_4_	−0.8912	2.254	0.192	1.76 (D)
Hex-H_2_O	−0.1923	2.458	−0.017	1.76 (D)
Hex-CO	0.1071	3.190	0.097	1.77 (D)
Hex-NH_3_	−0.5678	2.265	0.200	1.72 (D)

Adsorption energy E_a_, interatomic distance (*d*) between GaN monolayers and the molecule (Hydrazine (N_2_H_4_), water (H_2_O), carbon monoxide (CO), and ammonia (NH_3_)), electronic charge transfer Q on the molecule (positive values of Q mean charge transfer from the molecule to monolayer), and the electronic band-gap (I: indirect band gap, and D: direct band-gap).
