# Supplementary material for: GaN Haeckelite Single-Layered Nanostructures: Monolayer and Nanotubes
Source: Sci Rep. 2015 Dec 10;5:17902. doi: 10.1038/srep17902 (PMC4674713; doi:10.1038/srep17902)
Supplement: Supplementary Information [file srep17902-s1.pdf]

GaN Haeckelite Single-Layered Nanostructures: Monolayer and Nanotubes

*Dulce C. Camacho-Mojica, Florentino López-Urías**

Advanced Materials Department, IPICYT, Camino a la Presa San José 2055,
Col. Lomas 4a Sección, 78216, San Luis Potosí, México.

Figure S1: Dulce C. Camacho-Mojica et al.

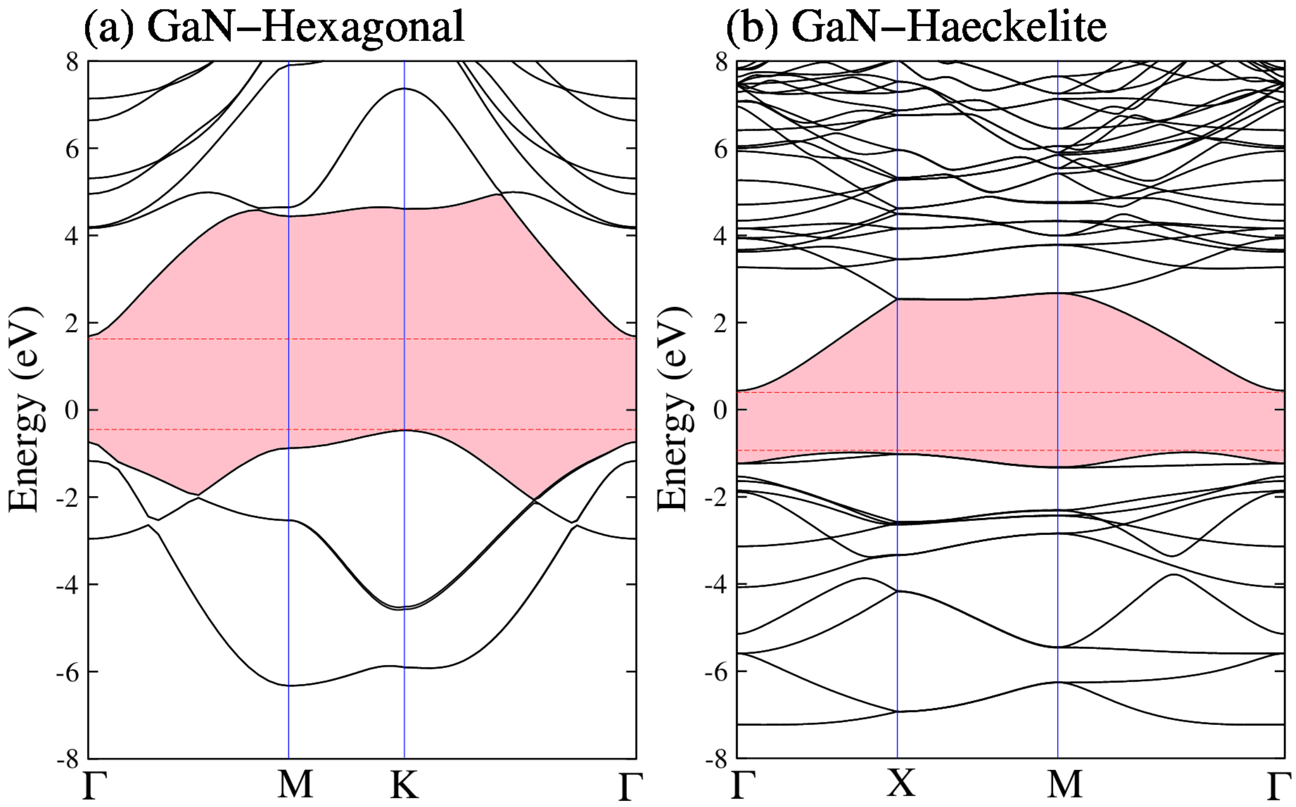

Figure S1: Spin-orbit coupling effects on the band-structure for the GaN monolayer. **(a)** GaN-hexagonal and **(b)** GaN-Haeckelite. Notice that in GaN monolayer, a band splitting energy in the valence band maximum at Γ-point is obtained. The calculations are carried out using the Quantum Expresso code. The Haeckelite monolayer exhibit non-significant changes in the valence bands when compared with the calculation without spin orbit coupling.

Figure S2: Dulce C. Camacho-Mojica et al.

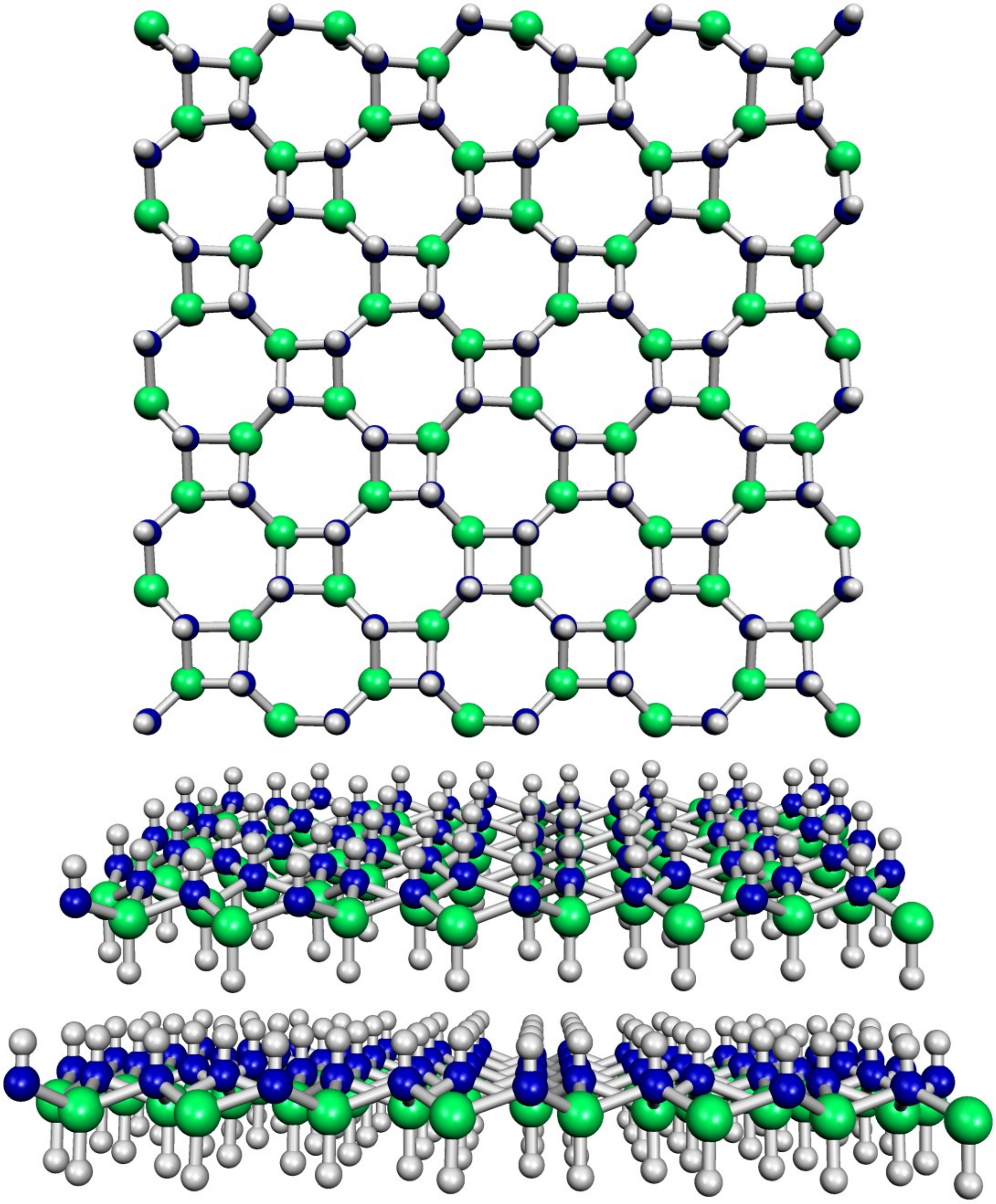

Figure S2: Different views of the relaxed structure of GaN monolayer passivated with hydrogen atoms (HGaNH).

Figure S3: Dulce C. Camacho-Mojica et al.

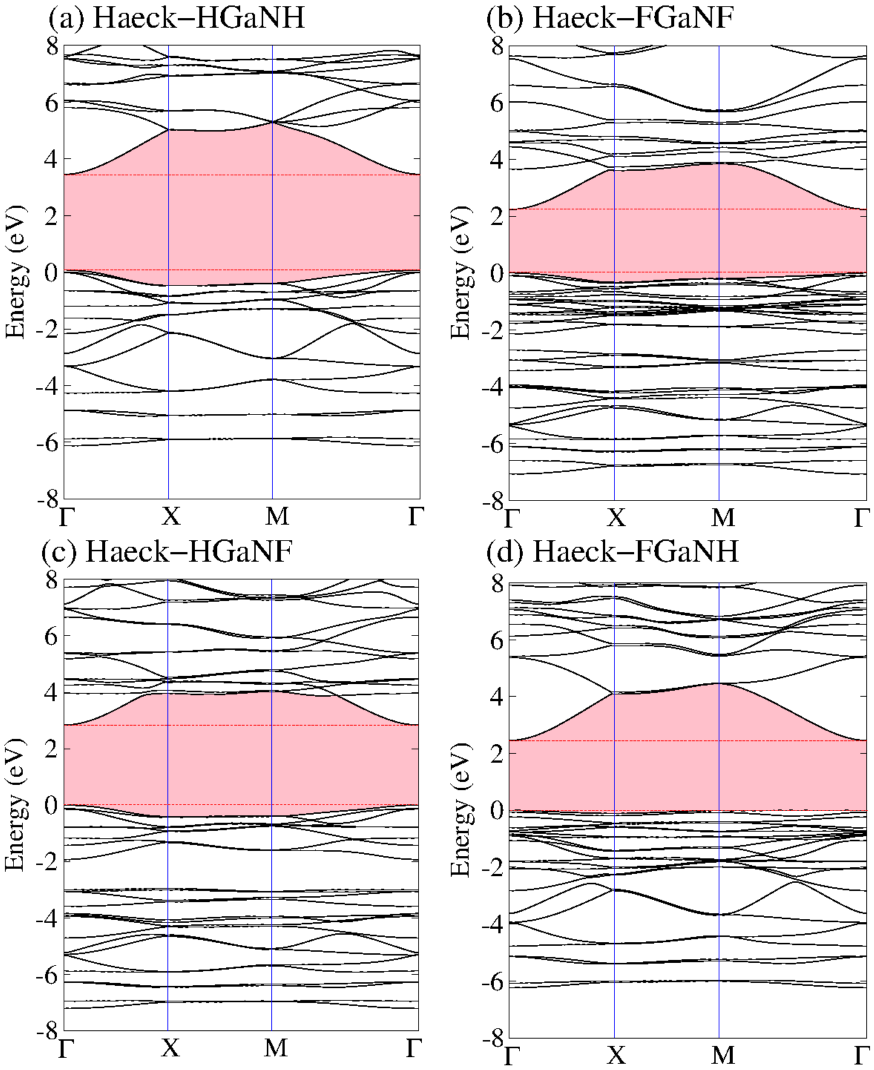

Figure S3: Band-structure calculations of two dimensional GaN Haeckelite structures passivated with H and F atoms

Figure S4: Dulce C. Camacho-Mojica et al.

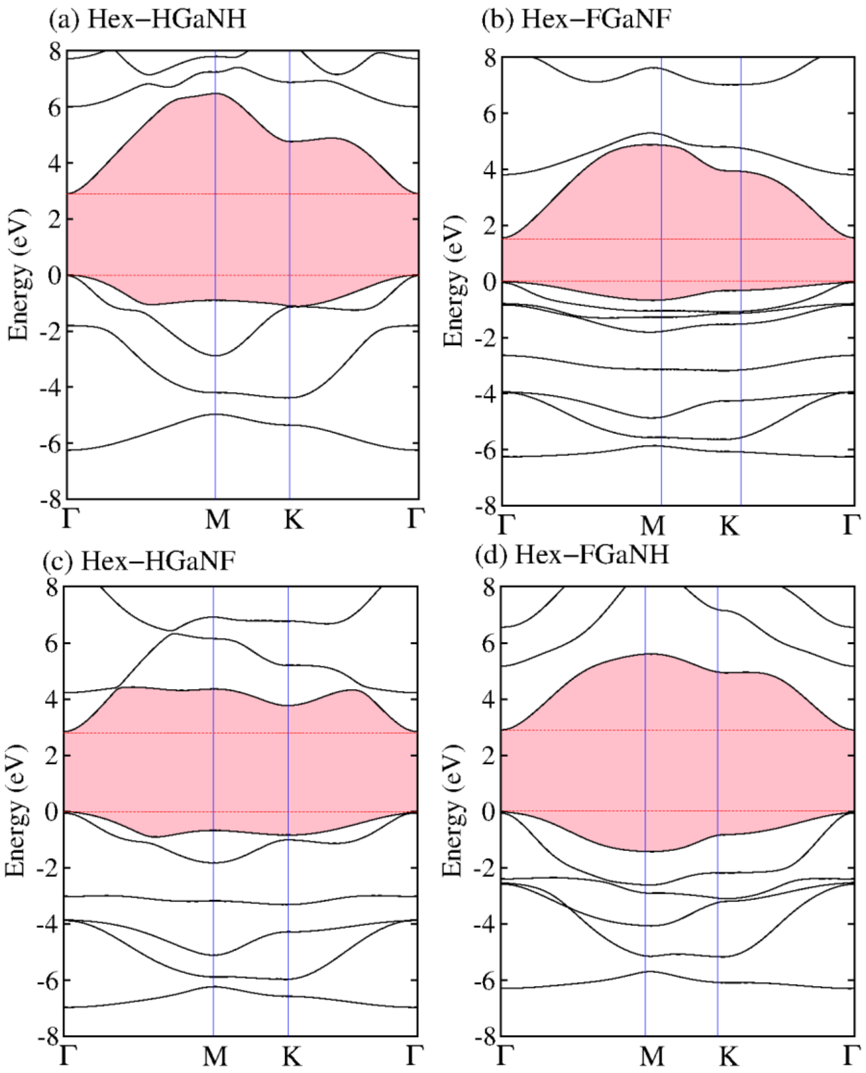

Figure S4: Band-structure calculations of two dimensional GaN Hexagonal structures passivated with H and F atoms
